# Intracerebral manifestation of iatrogenic, immunodeficiency-associated polymorphic B-LPD with morphology mimicking Hodgkin lymphoma: a case report and literature review

**DOI:** 10.1007/s12308-021-00478-0

**Published:** 2022-03-04

**Authors:** Leonie Saft, Marina Perdiki-Grigoriadi, Georgios Rassidakis

**Affiliations:** 1 Clinical Pathology and Cancer Diagnostics, Karolinska University Hospital and Institute, Solna, Stockholm, Sweden; 2 Clinical Pathology, University Hospital, Linköping, Sweden

**Keywords:** Polymorphic B-LPD, Iatrogenic, Lymphoproliferative disorder, Hodgkin-like cells, EBV, CNS

## Abstract

Iatrogenic immunodeficiency-associated lymphoproliferative disorders (IA-LPD) may arise in patients treated with immunosuppressive drugs for autoimmune disease or other conditions. Polymorphic EBV-positive B-lymphoproliferations often have features mimicking Hodgkin lymphoma and typically a self-limited, indolent course. We present an unusual case with isolated, intracerebral manifestation of polymorphic B-LPD with features of classic Hodgkin-lymphoma in an immunosuppressed patient treated with methotrexate and infliximab, including clinical-radiological features and a detailed description of morphological findings, together with a literature review on reported cases  of primary CNS manifestation of cHL and IA-LPD with Hodgkin-like morphology. The patient achieved complete remission following neurosurgery with gross total tumor resection and drug withdrawal without any additional treatment. Post-operative staging revealed no evidence for focal relapse or systemic disease during the 18 months follow-up period. Among the previously reported 24 cases of primary, isolated Hodgkin lymphoma in the central nervous system, three similar cases of iatrogenic, IA-LPDs were identified and are discussed here. Polymorphic B-LPD are destructive lesions with a range of morphologic features and disease manifestations. It is clinically important to recognize the spectrum of proliferations with features of classic Hodgkin lymphoma in immunodeficiency, iatrogenic settings, because they are likely to impact the choice of treatment strategies.

**Supplementary Information:**

The online version contains supplementary material available at 10.1007/s12308-021-00478-0.

## Introduction

Iatrogenic, immunodeficiency-associated lymphoproliferative disorders (IA-LPDs) are defined as lymphoid proliferations or lymphomas, arising in patients after long-term treatment with immunosuppressive drugs for an underlying autoimmune disease or other (non-hematological) disease in the non-transplant setting [1]. Methotrexate was the first reported immunosuppressive agent associated with iatrogenic LPD [2, 3]. Later on, cases of large B-cell lymphoma and classic Hodgkin lymphoma (cHL) were described in patients treated with TNF inhibitors [4–7]. The iatrogenic, immunodeficiency-associated LPDs constitute a spectrum of disease manifestations, including both cHL and LPDs with Hodgkin-like features. The latter likely represent EBV-positive B-cell LPDs and have similar features as the newly recognized EBV-positive mucocutaneous ulcer (EBVMCU), typically with a self-limited, indolent course [8].

We present a case of iatrogenic, immunodeficiency-associated, EBV-positive polymorphic LPD with morphology mimicking Hodgkin lymphoma in the CNS after long-term treatment with both methotrexate and infliximab (TNF inhibitor). The patient had no evidence of systemic disease and achieved complete remission after neurosurgery and drug withdrawal without any additional treatment. Among the previously reported 24 cases of primary, isolated CNS manifestation of HL, three additional cases of iatrogenic, immunodeficiency-associated EBV-positive LPD were identified and are discussed here.

## Clinical history

**Figure 1.**
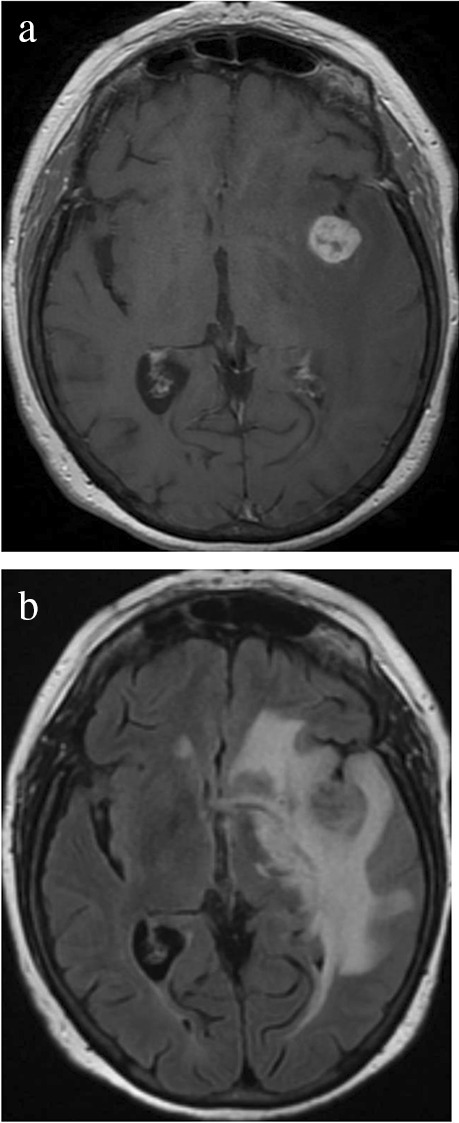
T1-weighted magnetic resonance image shows a left-sided, well-circumscribed contrast-enhancing lesion measuring 2 cm in diameter (**a**). T2-weighted FLAIR image (fluid attenuated inversion recovery) reveals marked surrounding edema (**b**)

A 75-year-old woman presented to the emergency department with acute onset of hemiparesis, impaired consciousness, and facial droop. Her past medical history included a diagnosis of neurosarcoidosis and long-term immunosuppressive treatment with methotrexate and infliximab (TNF inhibitor) for at least 6 years. A magnetic resonance imaging scan (MRI) of the brain revealed a solitary, well-circumscribed, contrast-enhancing, left-sided supratentorial lesion measuring approximately 2 cm with significant perifocal edema and mass effect (Fig.1). The patient’s neurological symptoms slightly improved following pre-operative corticosteroid administration. She underwent craniotomy for gross total resection of the lesion. The excised specimen consisted of several tissue fragments measuring up to 1.8 × 1.4 × 0.8 cm. Histological examination revealed a sharply demarcated, EBV-positive polymorphic infiltrate with Hodgkin-like cells, consistent with a diagnosis of iatrogenic, immunodeficiency associated lymphoproliferative disorder (IA-LPD). Methotrexate and infliximab were immediately withdrawn. An extensive staging work-up, including ultrasound and CT scan of the chest and abdomen, revealed no lymphadenopathy or other sign of systemic disease. Bone marrow examination was normal. Further extensive laboratory tests were reported unrevealing, but specific information on EBV serology testing was not available. The patient’s postoperative course was uneventful. A CT of the brain 6 and 12 months after surgery demonstrated only minor post-operative changes, and the patient achieved complete remission without any additional treatment. She died 18 months after initial presentation secondary to bacterial pneumonia and SARS-CoV2 infection.

## Materials and methods

The tissue was fixed in 4% buffered paraformaldehyde solution and embedded in paraffin. Tissue sections were stained with hematoxylin-eosin and Giemsa. Immunohistochemical stainings included CD3 (2GV6), CD20 (clone L26), CD15 (MMA), CD30 (Ber-H2), CD45 (RP2-18), CD68 (PG-M1), BOB-1 (SP-92), OCT-2 (MRQ-2), CD79a (SP-18), CD23 (SP-23), MUM-1 (EP190), PAX5 (SP34), p53 (DO-7), Ki67 (30-9), CD138 (clone B-A38) (all Roche/Ventana), kappa/lambda (polyklonal, Dako), and LMP-1 (CS.1-4, Dako) using an automated immunostaining system (Ventana Medical Systems). In situ hybridization for Epstein-Barr virus RNA (EBER transcripts) was performed on formalin fixed paraffin sections (FFPE) using the EBER-1 probe (Novocastra Laboratories, New castle upon Tyne, UK). PCR-analysis for immunoglobulin (IG) gene rearrangements was performed using genomic DNA isolated from FFPE tissue blocks (QIAGEN Tissue kit) and BIOMED-2 primers according to the BIOMED-2 PCR based protocol [9].

## Results

**Figure 2.**
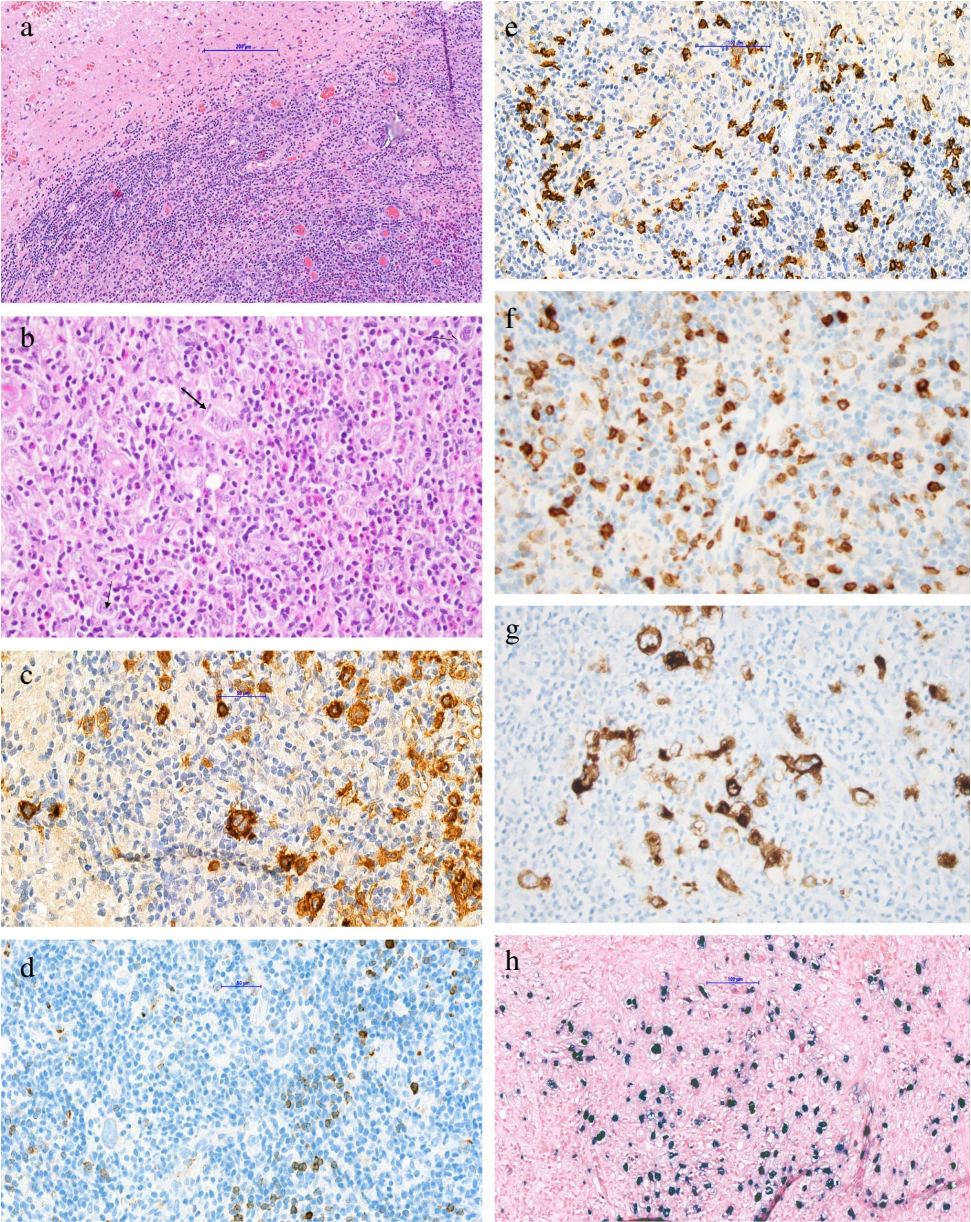
Histological examination demonstrates a well-demarcated, intracerebral polymorphic infiltrate with small lymphocytes, numerous plasma cells, eosinophilic granulocytes, and scattered large cells with Hodgkin/Reed-Sternberg morphology (**a,b**). The atypical cells stain positive for CD30 (**c**), negative for CD15 (**d**) and CD20 (**e**), CD79a weakly positive (**f**), and positive for both LMP-1 (**g**) and EBER/EBV (**h**)

Routine H&E staining demonstrated a well-circumscribed, intracerebral lesion consisting of a polymorphous infiltrate with a mixture of small lymphocytes, numerous polyclonal plasma cells, eosinophils, histiocytes and scattered large, atypical mono- and multinucleated, Hodgkin- and Reed-Sternberg-like cells (Fig. 2). The large cells stained positive for CD30, CD79a, OCT-2, PAX5, MUM-1, and p53, but mostly negative for CD20. The cells lacked expression for CD15, BOB-1, and CD45. In situ hybridization for EBER showed a range of EBV+ cells, including both large cells and numerous small lymphocytes. PCR analysis showed no evidence for immunoglobulin heavy chain gene rearrangement  (not shown). In the context of the combined clinical, morphological, and immunohistochemical findings and long-standing treatment with methotrexate and TNF inhibitor, a diagnosis of iatrogenic, immunodeficiency-associated EBV-positive polymorphic LPD with Hodgkin-like features was established.

### Review of the literature

Among the 24 published reports on primary, isolated CNS manifestation of cHL between 1980 and 2020 [10–33], three were diagnosed in patients with underlying autoimmune diseases and long-term immunosuppressive treatment. Therefore, these cases fall into the same WHO category of “other iatrogenic immunodeficiency-associated lymphoproliferative disorders” (Table 1) [18, 27, 28] . Two patients (66 and 47 years) with myasthenia gravis had been treated with azacytidine for 12 and > 20 years, and the other patient (74 years) had received long-term treatment with methotrexate and infliximab for rheumatoid arthritis. The lesions were intracerebral (frontoparietal lobe, cerebellum) and intraspinal, measuring between 1.4 cm and 3.5 cm in size. Complete surgical resection was achieved in two cases, while one patient with spinal engagement had diagnostic needle biopsies. Histology showed in all three cases a polymorphic infiltrate with scattered large, EBV+, and CD30+ HRS-like cells with variable expression of B-cell markers and negativity for CD15. PCR for heavy chain gene rearrangement indicated clonality in one case (no data available for the two other cases). Post-operative staging revealed no evidence for systemic disease in any of the patients. Two patients received additional radio-chemotherapy, while one patient was treated with radiotherapy only. All three patients were in complete remission at 3, 9, and 18 months follow-up, respectively. Therefore, the case presented here is the first reported case with complete remission without additional chemo- or radiation therapy.

**Table 1.** Isolated CNS manifestation of iatrogenic immunodeficiency-associated LPD with Hodgkin-like cells (from a literature review, Supplementary Table 1)

Author/year (ref)	Patient age/gender	Past medical history	Immunosuppressive drug	Location (size)	Diagnostic method	Histology (IHC)	EBV testing; molecular studies	Treatment	Outcome, time to last follow-up (months)
Herrlinger et al., 18 (18)	66/F	Myasthenia gravis	Azacytidine (12 years, 100 mg daily)	Left fronto-parietal	Surgical biopsy (15 mm)	HRS-like cells, CD30+, CD15-, CD20+, CD45-, EMA-, LMP+	EBER+ (range of positive cells); PCR indicated heavy chain gene rearrangement; no TCR-clonality	Surgery (complete resection). Post-op RT and chemotherapy (lomustine, procarbazine, vincristine)	CR, 18 months
Henkenberens et al., 27 (27)	47/M	Myasthenia gravis	Azacytidine (> 20 years)	Cerebellum; three separate lesions measuring up to 3.5 cm)	Surgical biopsy	HRS-like cells, CD30+, CD20 dim, CD79a dim, PAX5 dim, CD15-, LMP+ (restricted to large cells), EBNA2-	EBER+ (restricted to large cells); PCR not reported	Surgery (complete resection), post-op RT, chemotherapy (BEACOPP*)	CR, 9 months
Martinez et al., 28 (28)	74/F	Rheumatoid arthritis	Methotrexate, infliximab (long term)	Medulla oblongata; cerebellar peduncles; conus medullaris (1,4 cm)	Several core needle biopsies	HRS-like cells, CD30+; EBV-positive (not further specified)	PCR not reported	No complete resection. Post-op RT (no chemotherapy)	CR, 3 months
Our case	75/F	Neurosarcoidosis	Methotrexate, infliximab (> 7 years)	Left temporo-parietal (2x1 cm)	Surgical biopsy	HRS, CD30+, LMP-1+, CD45-CD15-, PAX5+, MUM-1+, CD20dim+/-, CD79a dim+, OCT2 dim+, BOB1-	EBER+ (range of positive cells); PCR negative for heavy chain rearrangement; no TCR-clonality	Complete resection; drug withdrawal. No adjuvant therapy	CR 17 months; Patient died secondary to bacterial pneumonia

*CR* complete remission, *HRS* Hodgkin/Reed-Sternberg, *RT* radiotherapy; *BEACOPP (bleomycin, etoposide, adriamycin, cyclophosphamide)

Among the other reported cases in the literature diagnosed as isolated cHL in the CNS (summarized in Suppl. Table 1), one patient had an underlying autoimmune hyperthyroidism (Graves’ disease) treated with radioactive iodine [17], and another patient had been treated with immunosuppressive drugs after a renal transplant [32], while the past medical history was unremarkable in all other reported cases.

## Discussion

We describe an unusual case of immunodeficiency-associated polymorphic B-cell LPD with cells mimicking Hodgkin cells which presented as a solitary lesion in the brain without evidence of systemic disease. The patient had a history of neurosarcoidosis and been treated with Methotrexate and Infliximab. Three similar cases with isolated CNS manifestation have been reported in immunocompromised patients, all with an indolent clinical course. In contrast to our case, these patients received combined radio-chemotherapy or radiotherapy only. Importantly, no evidence for systemic disease was detected during clinical follow-up. The described lesions had morphological features of classic Hodgkin lymphoma, e.g., presence of Hodgkin/Reed-Sternberg cells in an appropriate reactive/inflammatory background. Acknowledging the diverse overlapping histological patterns of cHL and EBV+ B-LPD with cells that mimic Hodgkin cells in the setting of iatrogenic immunodeficiency-associated LPD, a combination of clinical, morphological, and immunophenotypic findings is needed for reaching the correct diagnosis. For example, the lack of CD15-expression and variable expression of B-cell markers in CD30-positive HRS-like cells (all four cases) have been considered a feature against the diagnosis of cHL [34]. An additional useful parameter not typical for cHL is the wider range in cell size of the EBV-positive cells as highlighted by CD20, CD30, and EBER. EBV is almost always found in polymorphic LPD with Hodgkin-like features, including the newly recognized EBV-positive mucocutaneous ulcer [8]. Such cases have been described at different anatomic locations. In a more recent paper, Marcelis et al. describe a series of 72 biopsy-confirmed cases with immunomodulatory agent-related LPD of which the majority were non-Hodgkin lymphomas; cHL and polymorphic B-LPD represented 6 and 8 of the cases, respectively, and none of these presented as CNS manifestation. An association with EBV was seen in 75% of LPDs, with lower frequencies in other entities [35]. Given the clinical and histopathologic heterogeneity of IA-LPDs, the importance of adequate diagnostic material cannot be underemphasized [36].

## Conclusion

Iatrogenic immunodeficiency-associated LPD constitute a spectrum of disease manifestations, including EBV+ B-LPD with Hodgkin-like cells and cases that fulfil the criteria of classic Hodgkin lymphoma. The primary, isolated CNS manifestation of cHL is exceedingly rare [37–39]. Correct diagnosis is facilitated by consideration of the clinical history and the observed spectrum of EBV-positive cells. The importance of recognizing underlying immunodeficiency is crucial for appropriate treatment choice and remains a challenge for hematologists and oncologists. However, the high regression rate upon withdrawal of immunosuppressive therapy in some cases points to a condition for which a graded approach with conservative management is advised  in order to avoid unnecessary chemo-/radiotherapy.

## Supplementary Information

Below is the link to the electronic supplementary material.

Supplementary file1 (DOCX 27 KB)

## Data Availability

Not applicable.
