# Supplementary material for: Intracerebral manifestation of iatrogenic, immunodeficiency-associated polymorphic B-LPD with morphology mimicking Hodgkin lymphoma: a case report and literature review
Source: J Hematop. 2022 Mar 4;15(1):13–9. doi: 10.1007/s12308-021-00478-0 (PMC8895695; doi:10.1007/s12308-021-00478-0)
Supplement: Supplementary file 1 — Supplementary file1 (DOCX 27 KB) [file 12308_2021_478_MOESM1_ESM.docx]

**Supplemental Table 1.** Clinico-pathological features of cases reported as classic Hodgkin lymphoma with primary (isolated) manifestation in the CNS (literature review, 1980-2020)

| **Author/**  **Year (ref)** | **Patient age/ gender** | **Past medical history** | **Immuno-suppressive therapy** | **Location (size)** | **Histology (IHC)** | **EBV testing, Molecular studies** | **Treatment** | **Outcome, last follow-up (months/years)** |
| --- | --- | --- | --- | --- | --- | --- | --- | --- |
| Nagashima et al, 1980 (10) | 60/M | unremarkable | no | Falx cerebri | Mixed infiltrate with HRS-like cells | not reported | RT, Chemotherapy | died 12 months post-op; residual local tumor, no evidence for systemic disease (autopsy) |
| Bender & Mayernik, 1986 (11) | 34/M | unremarkable | no | Right frontoparietal, dural-based, extra-axial (5x3cm); cervical spine | Mixed infiltrate with HRS-like cells (OCT3+, Leu-M1-, CD45-) | not reported | Complete resection; RT, chemotherapy | CR, 12 months |
| Doorly et al, 1987 (12) | 51/M | unremarkable | no | Left cerebellum (2x2cm) | Mixed infiltrate with HRS-like cells (IHC not reported) | not reported | Complete resection; intrathekal Mtx; whole brain RT | CR, 12 months |
| Ashby et al, 1988 (13) | 62/M | unremarkable | no | Right fronto-temporal, well circumscribed, dural-based (4x4cm) | Mixed infiltrate, RS-cells (Leu-M1+, LN2+, CD45-) | not reported | Complete resection, RT, intrathekal methotrexate | CR, 14 months |
| Sickler et al, 1990 (14) | 84/F | unremarkable | no | Right parieto-occipital lobe, 2.5 cm, well-circumscribed | Mixed infiltrate, HRS-cells (CD30+, CD15-unsatisfactory; negative for B-/T-markers | not reported | Complete resection, RT | CR, 8 months |
| Clark et al, 1992 (15) | 53/ F | unremarkable | no | Right cerebellum | RS, nodular sclerosing | not reported | Complete resection, RT | CR, 6 months |
| Klein et al, 1999 (16) | 54/M | unremarkable | no | Right occipital lobe (2 x 1,5cm) | Mixed infiltrate with HRS cells, nodular sclerosis (CD30+ CD15+ CD45-, LMP1+, EMA, CD20+ <10%) | EBER+; PCR not reported | Complete resection, RT, chemotherapy | CR, 16 months |
| Biagi et al, 2000 (17) | 52/M | Graves hyperthyroidism | Radioactive iodine | Left temporo-parietal (1 cm) | Mixed infilrate with HRS cells (CD30+ CD15+, CD45-, CD20-, LMP1-) | EBER-; PCR not reported | Surgical resection; whole brain RT | CR, 21 months |
| *Herrlinger et al, 2000 (18) | 66/F | Myasthenia gravis | Azacythidine (12 yrs, 100 mg daily) | Left fronto-parietal lobe (1,5 cm) | Mixed infiltrate with HRS-like cells (CD30+, CD15-, CD20+, CD45-, EMA-, LMP1+) | EBER+ (range of positive cells); PCR indicated heavy chain gene rearrangement; no TCR-clonality | Complete surgical resection; post-op whole brain RT, chemotherapy | CR, 18 months |
| Johnson et al, 2000 (19) | 55/F | unremarkable | no | Tentorium cerebelli, dura-based, circumscribed (2x3cm) | Mixed infiltrate with RS, nodular sclerosis (CD30+, CD15(+), CD20-, LMP+) | not reported | Complete resection, whole brain RT | CR, 8 months |
| Heran et al, 2006 (20) | 42/M | unremarkable | no | Spinal, intradural extramedullary lesion (C5-T1) | Mixed infiltrate with HRS cells: CD30+ | not reported | Laminectomy, surgical biopsies; chemotherapy | CR, 4 years |
| De Castro et al, 2007 (21) | 63/M | unremarkable | no | Frontoparietal left; cerebellum (size not reported) | Mixed infiltrate with HRS-cells (CD30+, CD20+, CD15+, LMP1+) | not reported | Sterotactic biospy (no complete resection), post-op whole brain RT | NA |
| Hwang et al, 2007 (22) | 64/ F | unremarkable | no | Cerebellum, left (ill-defined, nodular) | Mixed infiltrate with HRS-cells (CD30+, CD45-, CD20-) | not reported | Complete resection; whole brain RT | CR, 16 months |
| Chotai et al, 2010 (23) | 24/M | unremarkable | no | Spinal, intradural extramedullary (lumbosacral region, 6x2 cm) | Mixed infiltrate with HRS-cells (CD30+; no other IHC markers reported) | not reported | Laminectomy, multiple biopsies (partial resection); chemotherapy | No CR (local residual tumor) |
| Foo et al, 2011 (24) | 58/ M | unremarkable | no | Left temporal lobe (size not provided) | Mixed infiltrate with HRS cells (CD30+, CD15+, CD45-, LMP1+) | not reported | Complete resection; whole brain RT | Initially CR; at 14 months CNS recurrence with multiple lesions; died 28 months after initial presentation |
| Kresak et al, 2013 (25) | 70/M | COPD, SCC (vocal cord) | no | Left cerebellum | Mixed infiltrate with HRS cells (CD15+, CD30+, CD45-, CD20-few cells positive) | not reported | Surgical resection; local RT (fossa posterior) | CR, 10 years |
|  | 72/F | unremarkable | no | Cerebellum (dural-based, size not provided) | Mixed infiltrate with HRS cells (CD15+, CD30+) | EBER+ | Surgical resection; focal RT | CR, 6 months |
| Gessi et al, 2013 (26) | 77/M | unremarkable | no | Cerebellum, left (6.5 cm) | HRS (CD30+, CD15+, MUM1+, LMP1+) | not reported | Complete resection; local RT | CR 6 months; MI at 9 months |
|  | 59/M | unremarkable | no | Brain stem (1 cm) | Mixed infiltrate with HRS (CD30+, MUM-1, LMP1+) | not reported | Complete resection | No follow-up data |
| *Henkenberens et al, 2014 (27) | 47, M | Myasthenia gravis | Azacythidine (>20 yrs) | Cerebellum; three separate lesions measuring up to 3.5 cm | HRS-like cells, (CD30+, CD20 dim, CD79a dim, PAX5 dim, CD15-, LMP1+ -restricted to large cells, EBNA2-) | EBER+ (restricted to large cells); PCR not reported | Surgery (complete resection), post-op RT, chemotherapy | CR, 9 months |
| *Martinez et al, 2014 (28) | 74, F | Rheumatoid arthritis | Methotrexate, Infliximab (long term) | Medulla oblangata; cerebellar peduncles; conus medullaris spinalis (1,4 cm) | Mixed infiltrate with HRS-like cells (CD30+; EBV-positive, not specified) | PCR not reported | No complete resection; post-op RT | CR, 3 months |
| Sharaf et al, 2014 (29) | 77/M | MGUS | no | Cerebellum, left (3x2 cm) | Mixed infiltrate with HRS (CD15+, CD30+, PAX5+, CD20+) | EBER+ | Complete resection, whole brain RT | CR, 7 months |
| Shivane et al, 2016 (30) | 59/F | unremarkable | no | Right frontal lobe  (3 x 2 cm, well-circumscribed) | Mixed infiltrate with HRS (CD30+, CD15+, MUM-1+, CD45-, LMP1+, variable expression of B-cellmarkers: CD20, CD79a, BOB1, OCT-2) | not reported  (IHC EBV+) | Surgery; chemotherapy, RT | CR, 2 months |
| Ahmad Alfaseh et al, 2019 (31) | 38/M | unremarkable | no | Cerebellum (left), (2x1cm) | Mixed infiltrate with HRS (CD30+, CD20-) | not reported | Complete resection; whole brain RT; chemotherapy | CR, 7 years |
| **Azriel et al, 2019 (32) | 49/M | Renal transplantation | yes (Prednisolon, cyclosporine, mycophenolate mofetil) | Intraventricular (septum pellucidum) | Mixed infiltrate with HRS (CD30+, CD15+, CD20+) | EBER+ | Incomplete resection; post-op chemotherapy | NA |
| Williamson et al, 2020 (33) | 48/F | Alopecia areata | Corticosteroids | Conus medullaris, cauda equina (13x14x22mm) | Mixed infiltrate with HRS-cells (CD30+, CD20+, PAX5+, MUM1+, CD45-) | EBER+ | Partly resection; local RT | CR, 10 months |

RT, radiotherapy; CR, complete remission; HRS, Hodgkin-/Reed-Sternberg; *Patient with underlying autoimmune disorder treated with immunosuppressive drugs (presented in detail, Table 1); **Post-transplant related lymphoproliferative disease (PTLD).
